# Morphometric profiling and EphB2/B4 and ephrin-B1 membrane protein expression analysis in vesicular, bulbourethral, and preputial glands in mice

**DOI:** 10.5455/javar.2026.m1047

**Published:** 2026-06-22

**Authors:** Md. Royhan Gofur, Mst. Suriya Tanjum Toma, Takayuki Nakajima, Takashi Tanida

**Affiliations:** 1 Department of Veterinary and Animal Sciences, University of Rajshahi, Rajshahi, Bangladesh; 2 Department of Veterinary Anatomy, Graduate School of Veterinary Science, Osaka Metropolitan University, Izumisano, Osaka, Japan

**Keywords:** Morphometry, histoarchitecture, membrane protein expression, sex glands, mice

## Abstract

**Objectives:** Male accessory sex glands are essential components of the reproductive system, providing secretions that support sperm function, fertility, and androgen-dependent reproductive processes. Despite the widespread use of mice as experimental models, comprehensive biometrical, histomorphometrical, and molecular characterization of their accessory sex glands remains limited. The present study investigated the vesicular, bulbourethral, and preputial glands of adult male mice with particular emphasis on biometric parameters, histological organization, and the expression of EphB2, EphB4, and ephrin-B1 membrane proteins.

**Materials and Methods:** Ten-week-old male ICR mice (*n* = 6) were used for morphometric profiling, RT-PCR, and immunofluorescence analyses.

**Results:** The vesicular glands showed highly folded mucosa lined by pseudostratified columnar epithelial cells and enclosed by well-developed smooth muscle layers. Bulbourethral glands were multilobular, composed of arborized acini lined by tall columnar epithelial cells, whereas preputial glands were modified sebaceous glands characterized by lobulated sebaceous acini with prominent basal cells. RT-PCR analysis revealed the presence of EphB4 and ephrin-B1 transcripts in all three glands, while EphB2 expression was detected only in the vesicular and bulbourethral glands. Immunofluorescence staining demonstrated that EphB4 and ephrin-B1 were predominantly localized in basal cells of vesicular and preputial glands and in secretory epithelial cells in the bulbourethral gland, with weaker expression in principal cells in the vesicular gland. Faint expression of EphB2, EphB4, and ephrin-B1 was observed in α-SMA–positive smooth muscle cells of vesicular and bulbourethral glands.

**Conclusions:** The findings provide the first integrated biometric and histomorphometric data and demonstrate distinct expression profiles of EphB2/B4 and ephrin-B1 membrane proteins in mouse accessory sex glands, suggesting their involvement in glandular structure and functional regulation.

## 1. Introduction

The male accessory sex glands are integral to the reproductive system, as they produce secretions that contribute to seminal plasma and thereby influence sperm function and fertility. In mice, the principal accessory reproductive glands include the vesicular (seminal vesicles), bulbourethral, prostate/coagulating, and preputial glands; each secretes distinct factors necessary for successful reproduction [1]. These secretions supply nutrients, enhance and stabilize sperm motility, aid in the formation of the copulatory plug, and modify the female reproductive tract to create a favorable environment for sperm survival and fertilization [2, 3]. The growth and function of the accessory sex glands are largely reliant on androgens, particularly testosterone and its metabolite dihydrotestosterone, making them reliable indicators in studies of endocrine function and reproductive toxicology [4]. For this reason, biometry of sex glands is often used as an indirect measure of circulating androgen levels or antiandrogen exposure [5]. Unlike humans, rodents possess a preputial gland, which plays a unique role in producing pheromones that mediate sexual and social behavior [6]. Taken together, the structure and secretory activity of the male accessory sex glands offer valuable insights into male reproductive physiology, androgen regulation, and mechanisms of toxicological response. Gofur [7] and Maruti et al. [8] reported the biometry and histomorphometry of accessory sex glands of bucks and buffalo bulls, respectively. However, there is no morphometric data available for mice.

Eph receptors and their ligand ephrins constitute a large family of membrane-bound proteins mediating direct cell–cell communication. As receptor tyrosine kinases, Eph receptors are categorized into EphA and EphB subclasses, which preferentially bind ephrin-A and ephrin-B ligands, respectively, although cross-class interactions can occur. Eph/ephrin engagement induces bidirectional signaling, comprising forward signaling through the receptor and reverse signaling through the ligand, mediated by receptor phosphorylation and activation of Src family kinases. This signaling system regulates key cellular processes such as adhesion, migration, and repulsion through actin cytoskeleton remodeling mediated by Rho-family GTPases [9, 10].

Recent observations have revealed that EphB/ephrin-B signaling is essential for the physiology and equilibrium of healthy adult organs and tissues, including maintaining homeostasis and forming epithelial boundaries in several epithelia, most likely in the skin, kidney, gut, stomach, and mammary glands [11, 12, 13]. In reproductive biology, certain ephrin-B ligands and EphB receptors have been associated with prostate cancer invasion and metastatic progression [14] as well as the growth of the prostate [15]. Recent investigations have identified the presence of ephrin-B and EphB proteins in epididymis and testis during both postnatal development and adulthood. Specifically, EphB4 and ephrin-B1 were detected in spermatogonia, macrophages, myoid cells, and Leydig cells, while EphB2 was observed in spermatozoa in testis [16, 17, 18]. In epididymal ducts, ephrin-B1 was localized to epithelium, whereas EphB4 and ephrin-B1 were found in smooth muscle cells surrounding the ductus epididymis [18, 19].

However, their expression and distribution within the male accessory sex glands remain largely unexplored. Based on the reported observations, it was hypothesized that EphB/ephrin-B members are also expressed in the vesicular, preputial, and bulbourethral glands in male mice and may contribute to their structural maintenance and functional regulation. On the other hand, the house mouse (*Mus musculus*) has long been a cornerstone of scientific research, serving as a vital model organism that has propelled countless breakthroughs in biology and medicine [20, 21]. The present study, therefore, focused on morphometric profiling and immunohistochemical EphB2/B4 and ephrin-B1 membrane protein expression analysis in these glands using a mouse model.

## 2. Materials and Methods

### 2.1. Ethical clearance

The experimental protocols were reviewed and approved by the Animal Research Committee of Osaka Metropolitan University (approval code: 25–058).

### 2.2. Animals

Ten-week-old ICR male mice (*n* = 6) were housed under standard laboratory conditions and used for biometric and histomorphometric evaluations, as well as for RT-PCR and immunofluorescence analyses. Animals were anesthetized with isoflurane (SN-487, Shinano Seisakusho Co., Ltd., Tokyo, Japan), after which the accessory sex glands (vesicular, preputial, and bulbourethral glands) were excised.

### 2.3. Biometric and histomorphometric analysis

The biometric study of vesicular, bulbourethral, and preputial glands was performed according to Aesha et al. [22]. Immediately after excision, the sex glands were preserved in 10% formalin. The formalin-fixed tissues were processed for histomorphometric analysis according to Gofur et al. [23]. Briefly, 5 µm thick paraffin-embedded sections of selected formalin-fixed glands were stained with routine hematoxylin and eosin stain according to Gofur et al. [24]. A compound microscope was used to closely examine the stained sections of sex glands. Using an oculometer (Erma, Japan) with a calibrated scale, the histomorphometrical parameters were measured (at 10x), and the photographs of these stained sections were taken using a photographic microscope system. All biometric and histomorphometric data were analyzed with the help of SPSS Statistics version 27.

### 2.4. RT-PCR analysis

Total RNA was isolated from the male accessory sex glands using Sepasol-RNA I Super G reagent (Nacalai Tesque Inc., Kyoto, Japan). First-strand cDNA was generated from 1 µg of RNA using M-MLV reverse transcriptase, RNase H (ReverTra Ace™, TOYOBO USA INC., NY, USA), and an oligo(dT)18 primer, according to the manufacturer’s protocol. For PCR amplification of EphB2, EphB4, ephrin-B1, and β-actin, 0.5 µl of the synthesized cDNA of the resulting 12.5 µl reaction mixture was used as a template with Taq DNA polymerase (ExTaq; TaKaRa Bio Inc., Otsu, Japan). The sequences of primers and the cycling conditions employed in the present study are summarized in Table 1. The PCR reaction was cycled using a TaKaRa PCR Thermal Cycler (TaKaRa Bio Inc., Otsu, Japan) and followed the cycling conditions: initial denaturation at 98°C for 60 sec, each cycle of denaturation at 98°C for 10 sec, annealing at the designed temperature for 30 sec, elongation at 72°C for 30 sec, and a final elongation at 72°C for 60 sec before resting at 4°C. PCR products were resolved in 1.5% agarose (Nacalai Tesque Inc., Kyoto, Japan) gels using GelRed^®^ Nucleic Acid Stain 10000X solution (Biotium Inc., Hayward, USA) for 20 min and visualized on a FAS-IV (NIPPON Genetics) transilluminator and gel documentation system.

**Table 1.** Primer pairs and cycle numbers used for PCR amplification.

Primer			Product size (bp)	Annealing temp. (°C)	Cycle number
EphB2	Forward	5´-CGA CGA GAA CAT GAA CAC TA-3´	583	53.0	35
	Reverse	5´-CCC GTT ACA GTA GAG TTT GA-3´			
EphB4	Forward	5´-AGC CCC AAA TAG GAG ACG AG-3´	540	57.9	35
	Reverse	5´-GGA TAG CCC ATG ACA GGA TC-3´			
Ephrin-B1	Forward	5´-TGC TTG ATC CCA ATG TAC TG-3´	520	55.0	35
	Reverse	5´-CGG AGC TTG AGT AGT AGG AC-3´			
β-Actin	Forward	5´-TCA TGA AGA TCC TGA CCG AG-3´	312	47.0	24
	Reverse	5´-GGT CTT TAC GGA TGT CAA CG-3´			

### 2.5. Antibodies

The details of antibodies used in the present study are presented in Table 2.

**Table 2.** Antibodies used for immunofluorescence staining.

Antibody	Host species	Catalog number	Working dilution	Conjugation	Vendor
Anti-ephrin-B1	Goat	AF473	1µg/ml	–	R&D Systems, Inc. (Minneapolis, MN, USA)
Anti-EphB2	Mouse	sc-130068	4µg/ml	–	Santa Cruz Biotechnology (Dallas, TX, USA)
Anti-EphB4	Goat	AF446	4µg/ml	–	R&D Systems, Inc. (Minneapolis, MN, USA)
Anti-α-SMA	Rabbit	ab5694	1:400	–	Abcam (Cambridge, UK)
Anti-goat IgG	Donkey	A11055	4µg/ml	Alexa Fluor 488	Molecular Probes, Inc. (Eugene, OR, USA)
Anti-mouse IgG	Goat	A11029	4µg/ml	Alexa Fluor 488	Molecular Probes, Inc. (Eugene, OR, USA)
Anti-rabbit IgG	Donkey	A21207	4µg/ml	Alexa Fluor 594	Molecular Probes, Inc. (Eugene, OR, USA)

### 2.6. Immunofluorescence staining

The male accessory sex glands were fixed in 10% formalin prepared with phosphate-buffered saline (PBS) for about 4 h at 4°C. Following fixation, tissues were rinsed in PBS with gentle agitation, cryoprotected overnight in 30% sucrose solution, and embedded in optimal cutting temperature (OCT) compound (Sakura Finetechnical Co., Ltd., Tokyo, Japan). Cryostat sections of 5–6 µm thickness were prepared and processed for immunofluorescence analysis. Double immunostaining was carried out as described previously [18]. In brief, sections were preincubated with 1% bovine serum albumin in PBS (BSA-PBS) and then exposed to primary antibodies at the following working concentrations: anti-EphB2 and anti-EphB4 (4 µg/ml), anti-ephrin-B1 (1 µg/ml each), and α-SMA (1:400 dilution). Incubations were performed for 90 min at 32°C in a humidified chamber. After PBS washes, slides were treated for 30 min at 32°C with secondary antibodies, which included a combination of Alexa Fluor 488-conjugated donkey anti-goat IgG or Alexa Fluor 488-conjugated goat anti-mouse IgG and Alexa Fluor 594-conjugated donkey anti-rabbit IgG (4 µg/ml each). After a final wash with PBS, tissue samples were mounted in a Fluoro-KEEPER antifade reagent, non-hardening type with DAPI (Nacalai Tesque), and imaged on an inverted fluorescence microscope (IX71; Olympus, Tokyo, Japan) equipped with a digital camera (DP72; Olympus) operated with the DP2-BSW software (Olympus).

## 3. Results

### 3.1. Morphometric profiling

The biometrical and histomorphometrical values of vesicular, bulbourethral, and preputial glands reflect the morphometric profiles of these glands in mice.

### 3.2. Biometry and histomorphometry of the vesicular gland in mice

**Figure 1.**
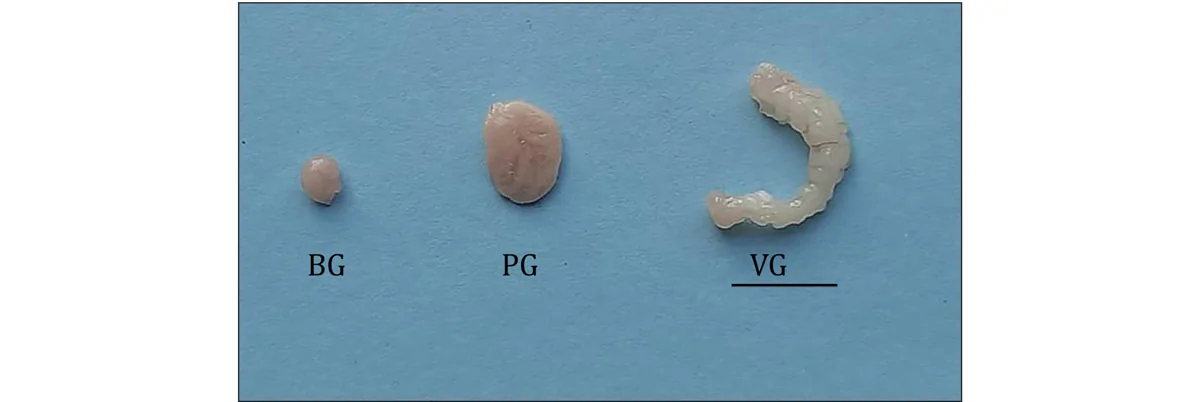
Gross images of the vesicular, preputial, and bulbourethral glands of mice. VG, vesicular gland (seminal vesicle); PG, preputial gland; BG, bulbourethral gland; scale bar, 1 cm.

**Figure 2.**
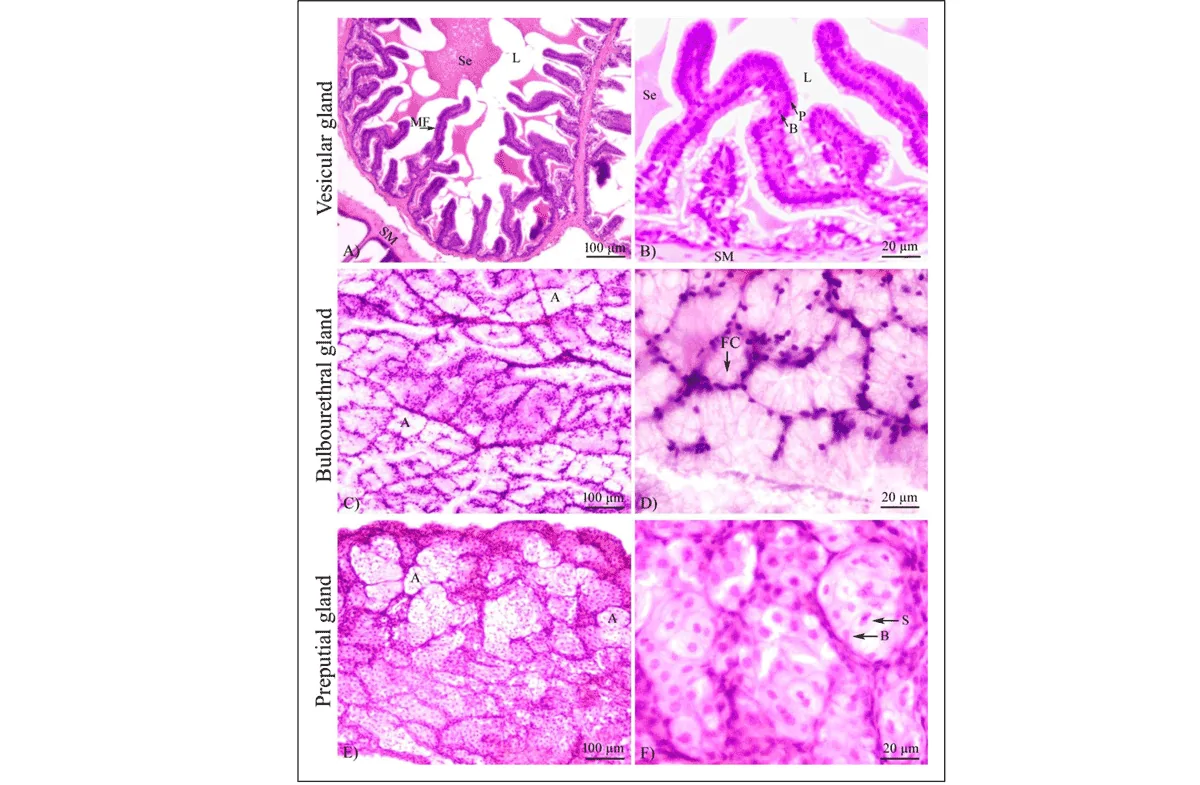
Photomicrographs illustrating the histological architecture of the vesicular, bulbourethral, and preputial glands of mice. Se, secretion; L, lumen; MF, mucosal fold; SM, smooth muscle; A, acinus; B, basal cell; P, principal cell; S, secretory cell; FC, foamy cytoplasm.

The seminal vesicles were paired, relatively large glands, comma-shaped and situated dorsolateral to the bladder and connected to the anterior prostate (coagulating gland) (Figure 1). The average biometric values of vesicular glands were presented in Table 3. Histologically, in mice, the seminal vesicle wall consisted of a mucosal layer, a lamina propria, and an underlying smooth muscle layer. The seminal vesicle had a highly folded mucosal lining of pseudostratified columnar epithelium containing tall columnar principal cells and smaller, triangular basal cells. The columnar epithelial cells exhibited prominent secretory granules and basally positioned nuclei. The mucosa forms extensive branching folds that give the lumen a honeycombed or lace-like appearance. Beneath the epithelial lining lies a delicate connective tissue layer, the lamina propria, composed of loosely arranged connective tissue cells and fibers, and it is encircled by smooth muscle cells. The glandular lumen was filled with strongly eosinophilic (pink-staining) secretory material (Figures 2A, B). In histomorphometry, the mean length of mucosal folds was 166.50 ± 23.85 µm, and the average length of principal cells was 13.86 ± 0.94 µm in mice’s vesicular glands.

**Table 3.** Biometric values (mean ± SE) of vesicular, bulbourethral, and preputial glands of mice.

Parameters	Length (cm)	Width (cm)	Weight (gm)
Vesicular gland	1.94 ± 0.03	0.51 ± 0.02	0.083 ± 0.003
Bulbourethral gland	0.49 ± 0.01	0.38 ± 0.01	0.019 ± 0.001
Preputial gland	1.02 ± 0.03	0.69 ± 0.02	0.055 ± 0.002

### 3.3. Biometry and histomorphometry of the bulbourethral glands in mice

The bulbourethral glands of mice (Figure 1) were positioned close to the base of the penis and were surrounded by the bulbocavernosus and ischiocavernosus muscles. Regarding the biometry, the mean values were presented in Table 3. Microscopically, the bulbourethral glands exhibited a multilobular structure. Each lobule consisted of glandular acini that drained into a central duct. The glands were embedded within dense connective tissue, and the acini were separated by a thin fibrous capsule. The glandular acini (secretory units) were highly arborized and lined by tall columnar epithelial cells with foamy cytoplasm and basally placed nuclei (Figures 2C, D). Regarding the histomorphometry, the secretory epithelial cells measured 16.14 ± 1.65 µm in height, and the secretory unit (acinus) was 51.67 ± 5.98 µm in diameter in bulbourethral glands of mice.

### 3.4. Biometry and histomorphometry of the preputial gland in mice

In mice, the preputial glands were specialized sebaceous glands. They were paired, large, flattened, tan-yellow structures situated subcutaneously on either side of the base of the penis (Figure 1). The mean biometric values of preputial glands were presented in Table 3. Histological analysis of the preputial glands revealed a lobulated structure enclosed by a dense connective tissue capsule. From this capsule, trabeculae extended inward, partitioning the gland into multiple sebaceous acini. Each acinus consisted of pale, foamy secretory cells with eosinophilic cytoplasm and dark-stained nuclei, surrounded by flattened, elongated basal cells. The basal cells around the acini were particularly prominent, and each acinus was supported by a fine network of connective tissue (Figures 2E, F). In the mouse preputial glands, the sebaceous acinus was 49.73 ± 4.63 µm in diameter.

### 3.5. EphB2/B4 and ephrin-B1 membrane protein expression analysis

The present study was intended to explore the expression array of EphB2, EphB4, and ephrin-B1 membrane proteins in vesicular, preputial, and bulbourethral glands in an adult mouse model using RT-PCR amplification and immunofluorescence staining.

### 3.6. RT-PCR analysis

**Figure 3.**
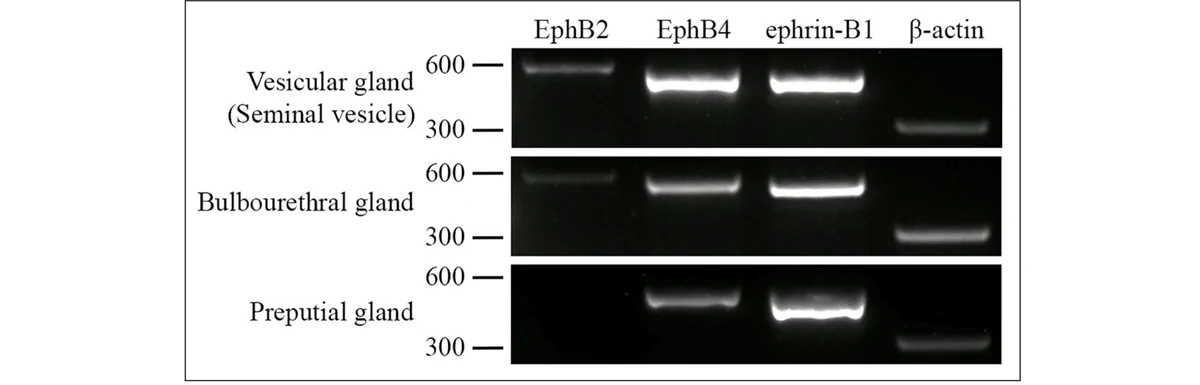
Gene expression of EphB2, EphB4, and ephrin-B1 in the vesicular, bulbourethral, and preputial gland of mice.

To detect EphB2, EphB4, and ephrin-B1 mRNA expression in mouse vesicular, bulbourethral, and preputial glands, we analyzed these glands by RT-PCR. We noticed transcripts for EphB4 and ephrin-B1 in vesicular, bulbourethral, and preputial glands, whereas EphB2 expressed only in vesicular and bulbourethral glands (Figure 3).

### 3.7. Immunohistochemical analysis

**Figure 4.**
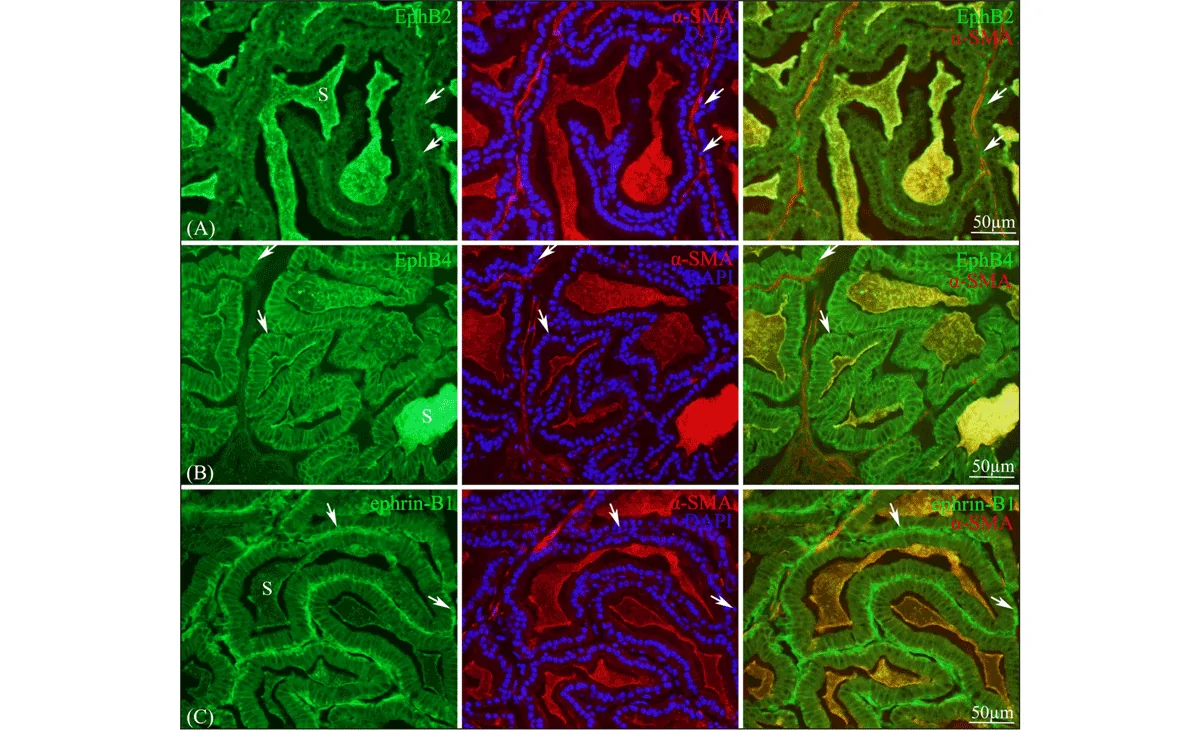
Immunofluorescence micrographs showing EphB2, EphB4, and ephrin-B1 localization in the vesicular gland of adult mice. (A) EphB2 is expressed weakly in basal cells, whereas EphB4 (B) and ephrin-B1 (C) are expressed both in principal and basal cells, but their expression intensity is higher in basal cells than principal cells (B and C). EphB2 and ephrin-B1 are faintly expressed in α-SMA-positive smooth muscle cells (A, C). S, intraluminal secretion; arrows indicate the basal cells.

**Figure 5.**
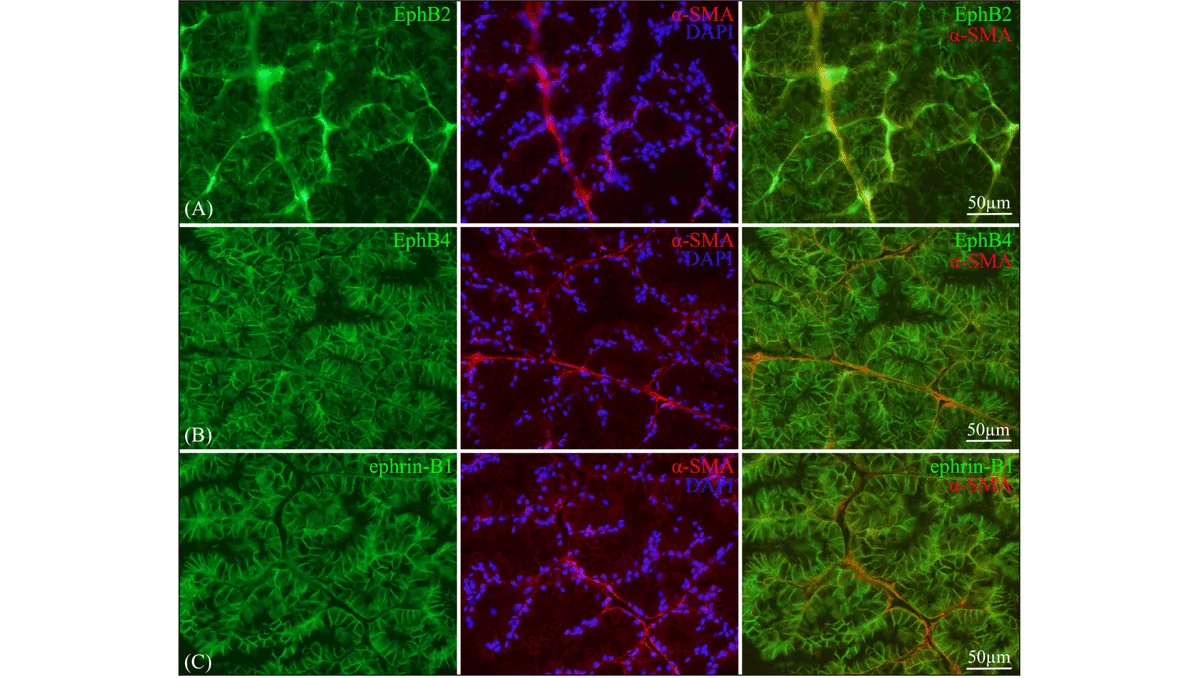
Immunofluorescence micrographs showing EphB2, EphB4, and ephrin-B1 localization in the bulbourethral gland of adult mice. EphB2 (A), EphB4 (B), and ephrin-B1 (C) are expressed in secretory epithelial cells, though EphB2 expression intensity is weak in epithelial cells. All these molecules are faintly expressed in α-SMA-positive smooth muscle cells (A – C).

**Figure 6.**
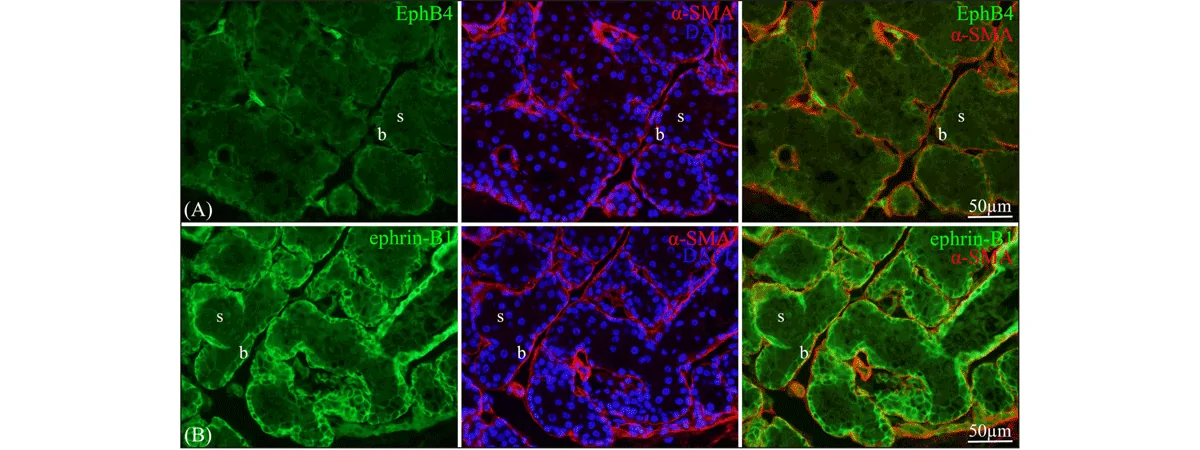
Immunofluorescence micrographs showing EphB4 and ephrin-B1 localization in the preputial gland of adult mice. EphB4 (A) and ephrin-B1 (B) are expressed only in basal cells, not in the secretory cells and α-SMA-positive smooth muscle cells. b, basal cells; s, secretory cells.

EphB4 and ephrin-B1 were localized in both principal and basal cells, but their expression intensity was higher in basal cells than principal cells, whereas EphB2 was expressed weakly in basal cells in the vesicular gland (Figure 4). EphB2 and ephrin-B1 were faintly expressed in α-SMA-positive smooth muscle cells in the vesicular gland (Figure 4). EphB2, EphB4, and ephrin-B1 were localized in secretory epithelial cells in the bulbourethral gland, though EphB2 expression intensity was weak in epithelial cells (Figure 5). All these molecules were also faintly expressed in α-SMA-positive smooth muscle cells in the bulbourethral gland (Figure 5). EphB4 and ephrin-B1 were localized only in basal cells, not in the secretory cells and α-SMA-positive smooth muscle cells in the preputial gland. Moreover, ephrin-B1 expression intensity was strong, and EphB4 expression intensity was weak in basal cells in the preputial gland (Figure 6).

## 4. Discussion

This study provides a concise anatomical, biometrical, histological, and histomorphometrical overview of the vesicular, bulbourethral, and preputial glands of adult mice, highlighting their distinct structural specializations. The vesicular glands were the largest glands and positioned in a similar anatomical position to bilateral seminal vesicles attached to a coagulating gland that was observed in rats [25] and in mice [1]. These glands showed extensive mucosal folding, pseudostratified columnar epithelium, and a well-developed smooth muscle layer, reflecting a high secretory capacity [26] and efficient expulsion of luminal contents. The histological architecture of a mouse seminal vesicle is similar to that of a rat [27, 28], donkey [29], and boar [30]. The lumen was filled with strongly eosinophilic (pink-staining) secretory material, the same as observed by Lima et al. [31] in bulls.

In mice, the bulbourethral glands are positioned close to the base of the penis, similar to their location in rats [32] and donkeys [33]. The biometric values of bulbourethral glands of adult mice in the present study were much smaller than those of the greater cane rat (0.24 ± 0.08 ml in volume, 0.63 ± 0.17 cm in diameter, and 0.19 ± 0.06 gm in weight), as reported by Adebayo et al. [32]. The histological architecture of mouse bulbourethral glands is similar to rat bulbourethral glands reported by Adebayo et al. [32]. Their compact organization and connective tissue framework suggest a role in producing mucous secretions [34] that facilitate urethral lubrication and protection during ejaculation. The acini in mice are lined by epithelium that resembles that of many other rodents and mammals [7, 35, 36]. The height of secretory epithelial cells and the acinus diameter were a little higher in mice in the present study than in the greater cane rat. In the bulbourethral glands of the greater cane rat, the secretory epithelial cells measured 14.15 ± 0.92 µm in height, and the diameter of the secretory units was 39.98 ± 3.86 µm [35]. This difference may be due to species differences.

The preputial glands are situated subcutaneously just lateral on either side to the base of the penis. Caro et al. [37] and Rajagopal et al. [38] observed the same location of this gland in mice and rats, respectively. The biometric values of preputial glands of adult mice in the present study were smaller than those of the adult male rat (1.42 ± 0.38 cm in length, 0.44 ± 0.11 cm in width, and 0.13 ± 0.01 gm in weight) that were observed by Rajagopal et al. [38]. The preputial glands exhibited a lobulated, sebaceous organization with foamy secretory cells and long excretory ducts, indicating adaptation for lipid-rich holocrine secretion rather than direct contribution to the ejaculate. The histological architecture of the preputial gland is similar, as observed by Homady and Majeed [6] and Rudmann et al. [39] in previous studies. Overall, the observed morphological diversity among these glands reflects functional specialization within the male accessory reproductive system of mice.

Eph-ephrin signaling modulates epithelial tissue homeostasis, integrity, and function in different organs like the skin, mammary glands, and intestine [11, 40]. Eph receptors and ephrins communicate through a multifaceted and dynamic interaction [41]. Because Eph receptors and ephrin ligands are tethered to cell membranes, direct cell-cell contact is typically required for signal initiation [40, 42]. Contact-dependent signaling occurs when Eph receptors and ephrins are expressed in different, complementary cell types. However, Eph receptor signaling complexes also play a significant role in cells where the receptor and ligand are co-expressed, especially within epithelial tissues [11, 43]. Recently it has been observed that EphB4 and ephrin-B1 co-express in spermatogonium, macrophages, myoid cells, and Leydig cells in the testis [17, 18]. In the present study, the co-expression of EphB4 and ephrin-B1 in both epithelial cell types of the vesicular gland suggests the possibility of autocrine or juxtacrine signaling that may contribute to epithelial homeostasis. The higher expression intensity observed in basal cells is particularly noteworthy, as basal cells are often regarded as progenitor or stem-like cells in glandular epithelia and play critical roles in epithelial renewal and differentiation [44, 45]. Enhanced EphB4–ephrin-B1 signaling in basal cells may therefore be associated with the maintenance of epithelial architecture or regulation of cell fate decisions [9, 13, 46].

EphB2, EphB4, and ephrin-B1 expression in secretory epithelial cells of the bulbourethral gland indicates that EphB/ephrin-B signaling may also participate in regulation of secretory gland function. The relatively weak EphB2 signal compared with EphB4 implies that EphB4 may act as the predominant receptor interacting with ephrin-B1 in this gland. Similar receptor-specific signaling preferences have been reported in exocrine and reproductive tissues, where EphB4–ephrin-B1 interactions regulate epithelial differentiation and tissue integrity [9, 13, 47]. The faint expression of these molecules in smooth muscle cells again supports a secondary, modulatory role in stromal compartments.

Notably, the preputial gland exhibited a highly restricted expression pattern, with EphB4 and ephrin-B1 localized exclusively to basal cells and absent from secretory epithelial and smooth muscle cells. The strong ephrin-B1 but weak EphB4 expression in basal cells suggests that ephrin-B1 may play a dominant role, potentially through reverse signaling mechanisms. Reverse ephrin-B signaling has been implicated in cytoskeletal remodeling, cell adhesion, and regulation of progenitor cell behavior [46, 48]. This restricted expression pattern highlights the functional heterogeneity of male accessory glands and underscores the context-dependent regulation of Eph–ephrin signaling.

## 5. Conclusions

This study provides the first comprehensive biometric and histomorphometric analysis as well as analysis of EphB2/B4 receptors and ephrin-B1 ligand expression in vesicular, bulbourethral, and preputial glands in adult mice. This study demonstrates the distinct structural specializations of these glands, reflecting functional specialization within the male accessory reproductive system of mice. This study also reveals distinct, gland-specific expression profiles of EphB2, EphB4, and ephrin-B1 in the vesicular, bulbourethral, and preputial glands, suggesting that EphB/ephrin-B signaling is involved in maintaining epithelial structure, regulating basal cell function, and mediating epithelial–stromal interactions. The predominance of EphB4–ephrin-B1 expression over EphB2 highlights a potentially central role for this receptor–ligand pair in accessory sex gland physiology and homeostasis. Further functional studies will be required to elucidate the precise mechanisms by which EphB/ephrin-B signaling contributes to gland development, maintenance, and secretory activity.

## Data Availability

The data presented in this study are available from the corresponding author upon reasonable request.

## List of abbreviations

Eph, erythropoietin-producing human hepatocellular; ephrin, Eph family receptor interacting; α-SMA, alpha-smooth muscle actin; ICR, Institute of Cancer Research; RT-PCR, reverse transcription–polymerase chain reaction; cDNA, complementary deoxyribonucleic acid; mRNA, messenger ribonucleic acid; μm, micrometer; μg, microgram; gm, gram; cm, centimeter; ml, milliliter; SE, standard error.
